# Verifying Family Authenticity in End‐of‐Life Care: A Case of Religious Refusal and Ethical Dilemma in an Older Adult With Dementia

**DOI:** 10.1111/ggi.70387

**Published:** 2026-02-18

**Authors:** Chisato Fujisawa, Toshiki Mizuno, Tomihiko Tajima, Yosuke Yamada, Kazuhisa Watanabe, Hirotaka Nakashima, Hitoshi Komiya, Hiroyuki Umegaki

**Affiliations:** ^1^ Department of Community Healthcare and Geriatrics, Graduate School of Medicine Nagoya University Nagoya Aichi Japan; ^2^ Department of Prevention and Care Science Research Institute, National Center for Geriatrics and Gerontology Obu Aichi Japan; ^3^ Motoyama Home Care Clinic Nagoya Aichi Japan; ^4^ Institute of Innovation for Future Society Nagoya University Nagoya Aichi Japan

Dear Editor,

In the terminal stage of illness, it is important to respect the patient's wishes, including those based on religious beliefs. However, in patients with advanced dementia, it can be difficult to confirm their wishes regarding end‐of‐life care. In such cases, healthcare professionals must often rely on family members or close acquaintances to infer the patient's previously expressed preferences. Nevertheless, if the family exploits the patient's cognitive impairment and uses the patient's assets or resources for purposes unrelated to care, how should healthcare professionals respond?

We encountered an elderly patient with advanced dementia who belonged to a religion that strictly prohibits certain medical interventions. The family of the patient, who lived with her, refused all medical treatments, claiming that they were honoring her wishes. However, they later abandoned her care. Ultimately, it was confirmed that they were her legal family members; however, they were also strongly suspected of being affiliated with the same religious group. This case highlights the need to protect terminally ill older adults from exploitation by verifying the authenticity of individuals claiming to be family members.

A Japanese woman in the late 80s presented to a medical facility with loss of appetite, fever, and impaired consciousness lasting 1 week. Body temperature was 38.0°C, blood pressure was 90/60 mmHg, and pulse rate was 90 beats per minute. Urinalysis revealed 4+ leukocytes, and abdominal computed tomography demonstrated renal enlargement with perirenal inflammation. Laboratory findings showed severe dehydration (white blood cell count, 14 000/μL; C‐reactive protein, 10 mg/dL; blood urea nitrogen, 60 mg/dL; creatinine, 6.0 mg/dL). Pyelonephritis was suspected, and antibiotic and fluid replacement therapy was recommended.

However, the two individuals accompanying the patient, who claimed to be her family members, refused hospitalization and all treatment for religious reasons. They stated that their purpose in visiting the hospital was “to confirm how much longer she might live.” The patient's Mini‐Mental State Examination score was 0, and she was unable to communicate. According to their account, she had always refused medical procedures and medications for religious reasons when she had been conscious and oriented. After consultation with the social worker and the hospital's medical safety department, the decision was made to respect the patient's presumed religious beliefs as claimed by the family. However, no one verified whether the two individuals were, in fact, her blood relatives.

The two individuals initially opposed arranging a home‐visit physician, citing financial concerns, but eventually relented. Although home‐visit nursing services were recommended, they declined. After the patient returned home, the two individuals continued to refuse antibiotic and fluid replacement therapy, so the home‐visit physician planned only weekly visits. At home, the patient did not appear to be properly cared for—she developed deep pressure ulcers, her hygiene was not maintained, and she was barely provided with food. Despite repeated explanations about wound care and personal hygiene, the two individuals did not comply. In response to this situation, the home‐visit physician developed concerns about whether the wishes asserted by the family truly reflected the patient's original intentions and therefore reported the case to the municipal authorities.

Following a report by the home‐visit physician to the municipal authorities, an investigation confirmed that the two accompanying individuals were family members; however, it strongly suggested that they were also affiliated with the same religious group. It was also revealed that the household had previously been reported by neighbors to municipal authorities for concerns related to living conditions, including hoarding, and had subsequently been monitored. The patient died 2 weeks after discharge. Some religions require monetary donations from followers, and in this case, it could not be ruled out that the patient was a victim of such religious exploitation. The clinical course is summarized in the Figure 1.

**FIGURE 1:**
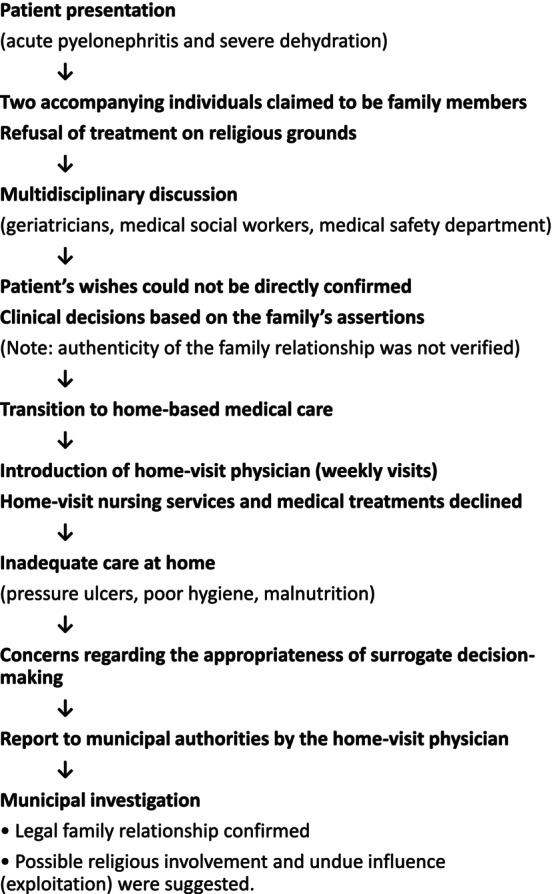
Clinical course and clinical management in the present case.

This case highlights two important issues:

1. To what extent, in clinical practice, should clinicians verify whether those claiming to be family members are in fact the patient's relatives?
2. When patients lack the cognitive function to express their will, how can health care professionals determine whether the “patient's wishes” asserted by family members genuinely reflect the patient's own will?

These questions emphasize the difficulty and importance of verification in the care of vulnerable older adults. Previous studies have identified identity, trust, and relationships as central to clinical ethics and patient safety, [1, 2] yet no study to date has addressed how to confirm the authenticity of family members involved in decision‐making. In actual practice, it is rare to verify relationships through documentation such as family registers, legal guardianship papers, or advance directives. However, in an aging society where neglect, abuse, or crime cannot be excluded, confirming family legitimacy and the authenticity of reported patient wishes represents an ethical necessity.

This case demonstrates the importance of early multidisciplinary collaboration and timely involvement of social welfare services when concerns arise regarding the appropriateness of surrogate decision‐making and caregiving in patients with advanced dementia. In the present case, no clear signs of abuse were evident at the initial stage; however, clinical deterioration during home‐based care subsequently raised concerns about the validity of surrogate decisions. When even a slight suspicion of abuse or neglect arises, early reporting to municipal authorities and proactive use of community‐based support resources are essential to facilitate assessment of decision‐making appropriateness and to ensure closer alignment with the patient's best interests.

Verifying family identity should not be viewed as distrust but rather as a safeguard intended to protect cognitively impaired older adults from exploitation disguised as familial or religious authority. Establishing verification procedures and promoting multidisciplinary discussion—including medical, legal, and social perspectives—may help to ensure that decisions attributed to “the patient's will” genuinely reflect the patient's wishes and preserve their dignity.

## Funding

This work was supported by grants from the Hori Sciences and Arts Foundation, the Research Grants on Aging and Geriatric Medicine awarded by the Japan Geriatrics Society, and the Kowa Foundation for Life Science Promotion. These funding bodies had no role in the study design, interpretation of data, or writing of this report.

## Consent

According to the response from the Ethics Committee of Nagoya University, obtaining the patient's consent is fundamentally required. However, in this case, the patient had already passed away, and it was uncertain whether the accompanying individual who claimed to be a family member was truly a relative; therefore, consent could not be obtained. Nevertheless, in situations where obtaining consent is not feasible, it is acceptable to publish information that may contribute to medical and scientific advancement, provided that maximum care is taken to ensure that no individual can be personally identified. In this case report, we have made every possible effort to anonymize all medical information so that neither the patient nor any family member can be identified.

## Data Availability

The data that support the findings of this study are available on request from the corresponding author. The data are not publicly available due to privacy or ethical restrictions.
